# Impact of child malnutrition on the specific anti-*Plasmodium falciparum *antibody response

**DOI:** 10.1186/1475-2875-8-116

**Published:** 2009-06-02

**Authors:** Florie Fillol, Jean Biram Sarr, Denis Boulanger, Badara Cisse, Cheikh Sokhna, Gilles Riveau, Kirsten Bork Simondon, Franck Remoué

**Affiliations:** 1 Institut de Recherche pour le Développement, Unité mixte de Recherche 145, 911 Avenue Agropolis, BP64501, 34394 Montpellier, France; 2 ONG Espoir Pour La Santé (EPLS), BP 226 Saint-Louis, Sénégal; 3 Université Cheikh Anta Diop, Department of Parasitology, Dakar, Senegal; 4 Institut de Recherche pour le Développement, Unité mixte de Recherche 198, Route des Pères Maristes, BP 1386, 18524 Dakar, Sénégal; 5 Institut de Recherche pour le Développement, Unité de recherche 016, 911 avenue Agropolis, BP64501, 34394 Montpellier, France

## Abstract

**Background:**

In sub-Saharan Africa, preschool children represent the population most vulnerable to malaria and malnutrition. It is widely recognized that malnutrition compromises the immune function, resulting in higher risk of infection. However, very few studies have investigated the relationship between malaria, malnutrition and specific immunity. In the present study, the anti-*Plasmodium falciparum *IgG antibody (Ab) response was evaluated in children according to the type of malnutrition.

**Methods:**

Anthropometric assessment and blood sample collection were carried out during a cross-sectional survey including rural Senegalese preschool children. This cross-sectional survey was conducted in July 2003 at the onset of the rainy season. Malnutrition was defined as stunting (height-for-age <-2 z-scores) or wasting (weight-for-height <-2 z-scores). The analysis was performed on all malnourished children in July (n = 161, either stunted, n = 142 or wasted, n = 19), pair-matched to well-nourished controls. The IgG Ab response to *P. falciparum *whole extracts (schizont antigens) was assessed by ELISA in sera of the included children.

**Results:**

Both the prevalence of anti-malarial immune responders and specific IgG Ab levels were significantly lower in malnourished children than in controls. Depending on the type of malnutrition, wasted children and stunted children presented a lower specific IgG Ab response than their respective controls, but this difference was significant only in stunted children (P = 0.026). This down-regulation of the specific Ab response seemed to be explained by severely stunted children (HAZ ≤ -2.5) compared to their controls (P = 0.03), while no significant difference was observed in mildly stunted children (-2.5 < HAZ <-2.0). The influence of child malnutrition on the specific anti-*P. falciparum *Ab response appeared to be independent of the intensity of infection.

**Conclusion:**

Child malnutrition, and particularly stunting, may down-regulate the anti-*P. falciparum *Ab response, both in terms of prevalence of immune responders and specific IgG Ab levels. This study provides further evidence for the influence of malnutrition on the specific anti-malarial immune response and points to the importance of taking into account child malnutrition in malaria epidemiological studies and vaccine trials.

## Background

Children under five years of age are particularly vulnerable to *Plasmodium falciparum *infection. Each year, about 800,000 children die of malaria, and 75% of these deaths occur in African children [1,2]. Moreover, undernutrition is highly prevalent in developing countries and is considered to be the underlying cause of more than 50% of all childhood deaths in the world [3]. In sub-Saharan Africa, 38% of children under five years of age suffer from chronic malnutrition or stunting (height-for-age z-score below -2 of an international growth reference), and acute malnutrition or wasting (weight-for-height z-score below -2) affects 9% of preschool children [1]. The interaction between malaria and malnutrition has been investigated for many years. It is now widely recognized that malnutrition and malaria share certain consequences, including cognitive impairment and decreased school performance [4-6]. Although numerous studies have shown a deleterious effect of malaria on nutritional status [7-9], whether and how malnutrition influences malaria morbidity remain unknown. Several older studies based on hospital admissions for severe malaria showed lower risk of malaria infection among undernourished children [10-12]. However, results of recent community-based studies are conflicting: two studies showed that stunting increased the risk of malaria morbidity among rural children in Gambia [13,14], whereas a trial in Papua New Guinea indicated that stunting protected children from *P. falciparum *malaria [15]. In addition, several studies found no significant association between stunting or height-for-age z-score and malaria morbidity [16-20]. With regard to wasting, some studies showed a trend to lower malaria-related morbidity among wasted children [13,18,21]. Altogether, these studies point to the importance of taking into account the kind of child malnutrition (stunting/wasting) in the relationship between malaria and malnutrition.

From July to December 2003, an observational follow-up study was conducted in a cohort of 2–59-month-old children living in a rural area of Senegal where malaria transmission was highly seasonal. The influence of child malnutrition at the onset of the rainy season upon subsequent susceptibility to malaria was investigated during that survey [20]. Results indicated that wasted children were at lower risk of experiencing at least one subsequent clinical malaria attack, whereas no association was observed in stunted children. However, among parasitaemic children in July 2003, stunted children had a significantly greater risk of being highly parasitaemic. Some non-biological explanations were considered to account for these unexpected results, such as overprotection of wasted children by their mothers. It was also assumed that the influence of child nutritional status on subsequent malaria morbidity may be related to modulation of malaria immunity. There is now clear evidence that malnutrition down-regulates immune functioning, resulting in higher risk of infection [22,23]. Some studies have shown that micronutrients are significant modulators of anti-pathogen immunity [24,25]. However, very few studies have investigated the relationship between malaria, specific immunity and malnutrition [15,26-28]. Among these studies, two showed no impact of malnutrition on the antibody (Ab) response to *P. falciparum *[27,28], whereas one survey indicated lower specific Ab levels in children who suffered from malnutrition [26]. In addition, a trial in Papua New Guinea showed that the IgG Ab response to *P. falciparum *schizont extract was lower in wasted children than in well-nourished children [15].

These discrepant results demonstrate the need to further investigate the impact of child malnutrition on anti-malarial immunity. Thus, a first approach was to determine whether child malnutrition modulates the overall anti-*P. falciparum *immune response. It has been previously shown that Ab levels directed to wide antigens of *P. falciparum*, such as schizont antigens, were associated with *P. falciparum *infection [29] and, therefore, represented an overall view of anti-malarial immunity [30,31]. Consequently, to investigate the impact of child malnutrition on the overall anti-*P. falciparum *immune response, IgG Ab levels specific to *P. falciparum *whole extracts (schizont antigens) were assessed according to child malnutrition, stunting and/or wasting and the severity of stunting.

## Methods

### Study area and population

The study was performed in Niakhar, a rural district of Senegal located 150 km southeast of the capital city of Dakar. The rainy season occurs from early July to October. No river and few permanent waterholes are found in the area. Consequently, *P. falciparum *malaria transmission is highly seasonal and takes place from August to October, with an average of 9–12 infective bites per person per year [32]. The mortality rate for children aged 1–4 years was 144 per 1000 live-births from 1994–1999 [33]. Malaria accounts for a quarter of all deaths in 1–4-year-old children [34]. The nutritional status of infants and preschool children is largely dependent on season, with the greatest weight-for-height and height-for-age at the end of the dry season (March-May) and the lowest averages following the end of the rains (October-November) [35].

### Study design

The present study is a nested case-control study among a cohort of 984 preschool children living in eleven villages in the area. This cohort was followed during one malaria transmission season from July to December 2003. The study design has been described in detail elsewhere [20]. Briefly, an intermittent preventive treatment (IPT) trial was conducted in 2002 among 2–59 month-old rural Senegalese children [36]. To assess the risk of malaria attack after terminating the IPT intervention, a longitudinal observational follow-up survey was conducted from July to December 2003 among children previously included in the IPT trial. A cross-sectional survey was conducted in July upon inclusion at follow-up to collect anthropometric data and blood samples.

The present analysis is a cross-sectional sub-study using data collected in July at the onset of the follow-up survey.

### Participants

Subjects included in this analysis represent a sub-sample of the cohort followed in 2003.

Inclusion criteria for children included in the 2003 follow-up survey have been previously described [20]. Briefly, 984 preschool children baseline included in the IPT trial in 2002 were enrolled in July 2003 and followed until December 2003. Among the 984 children enrolled in July, 874 were included in an analysis which investigated the influence of child malnutrition on subsequent malaria morbidity [20].

Among the 874 children, those eligible for the present analysis were all stunted children (n = 165) and wasted children (n = 27) in July 2003. Children both stunted and wasted (n = 8) were excluded from the present analysis which aims at investigating the influence of the type of malnutrition on anti-malarial Ab response. Fifteen children were absent at the time of blood sample collection or refused blood sample and were excluded from the analysis. The nutritional status and the age of these excluded children were not statistically different from those of the included children.

The 161 remaining malnourished children (142 stunted and 19 wasted) were randomly pair-matched to well-nourished control children belonging to the cohort. Finally, a total of 322 children were included in the present analysis.

### Ethics

Both the 2002 and 2003 anti-malarial studies and the present study followed ethical principles according to the Helsinki Declaration and were approved by the ethical committees of the Ministry of Health of Senegal (August 2002 and May 2003, respectively) and the IRD (January 2004). The anti-malarial trial was approved by the ethical committee of the London School of Hygiene and Tropical Medicine in June 2002. Informed consent was obtained from the study population.

### Parasitaemia assessment

Capillary blood samples were collected in July. Parasite densities were estimated in thick blood films, assuming an average white-blood-cell count of 8,000 per μL as previously described [20]. All slides related to acute episodes were read by two laboratory technicians. Among the 322 children included in this analysis, 16 cases (malnourished) and 14 controls did not undergo parasite density assessment due to technical failure during the processing of the slides.

### Anthropometric measurements

Anthropometric data were collected during home visits by two trained measurers in July 2003 [20], in accordance with internationally recommended procedures [37]. Weight measurements were taken using baby scales (SECA, Hamburg, Germany), precise to the nearest 10 g, for children weighing less than 16 kg, and an electronic scale (Téfal, Paris, France), precise to the nearest 100 g for older children. Recumbent length measurements were taken for children under 2 years of age, while standing height was measured beyond that age. Measurements were precise to the nearest mm. Height and length measurements were taken twice at each visit and the average was used for analysis.

### Evaluation of the IgG Ab response

The IgG Ab levels directed to total schizont antigen were assessed in sera of included children using antibody capture ELISA. Total schizont antigen is a soluble extract of *P. falciparum *schizont lysate obtained from infected erythrocytes and kindly provided by A-M. Schacht from the Pasteur Institute of Lille.

Schizont extracts (2 μg/ml) were coated on flat-bottom microtiter plates (Nunc, Roskilde, Danemark) with 100 μL/well for 2 h 30 at 37°C. Plate wells were then blocked for 30 min at room temperature with 200 μL of blocking buffer, pH 6.6 (phosphate-buffered saline, PBS), 0.5% gelatin (Merck, Darmstadt, Germany) and washed one time with PBS, pH 7.2, 0.1% Tween 20 (Sigma, Saint Louis, MO, USA). Individual sera were incubated in duplicate at 4°C overnight at a 1/100 dilution (in PBS-Tween-0.1%). This dilution was determined as optimal after several preliminary experiments [29]. For detecting human IgG, plates were incubated for 90 min at 37°C with 100 μL of mouse biotinylated mAb to human IgG (BD Pharmingen, San Diego CA, USA) diluted 1/1000 in PBS-Tween 0.1%, after washing three times with PBS-Tween 0.1%. Plate wells were then washed four times with PBS-Tween and incubated for 30 min at room temperature with 100 μL of peroxidase-conjugated streptavidin (Amersham Biosciences, les Ulis, France). After washing six times with PBS-Tween, colorimetric development was carried out using ABTS (2.2'-azino-bis (3-ethylbenzthiazoline 6-sulfonic acid) diammonium; Sigma) in 50 mM citrate buffer (Sigma, pH = 4, containing 0.003% H2O2), and absorbance (optical density, OD) was measured at 405 nm. Individual results were expressed as ΔOD value calculated according to the formula: ΔOD = ODx-ODn, where ODx was the individual OD value and ODn was the individual OD value for each serum without antigen. Negative controls (European individual, n = 30) were used for each assay (ODneg). A subject was considered as an immune responder if his ΔDO was higher than the ODneg arithmetic mean + (3 × SD) value.

### Statistical analyses

The nutritional indicators height-for-age (HAZ) and weight-for-height (WHZ) were computed in z-scores of the WHO/NCHS reference using Epi Info software V.6. Stunting and wasting were defined for values below -2 for HAZ and WHZ, respectively. Mild stunting was defined as -2.5 < HAZ <-2.0 and severe stunting as HAZ ≤ -2.5. In literature the commonest threshold used to define severe stunting is HAZ ≤ -2.5. However, the use of thresholds for malnutrition is often debated as these thresholds are arbitrary [38]. Moreover the -2.5 z-scores cut-off was used in this study to provide a sufficient number of severely stunted subjects in order to perform analyses with enough statistical power. Upon assessment in July 2003, children were allocated to one of five age strata: 12–23.9, 24–35.9, 36–47.9, 48–60 and over 60 months, respectively.

Each malnourished child (either stunted or wasted) was pair-matched to a control based on age strata, sex and place of residence. Malnourished children were randomly pair-matched to control children using a random table number. A control child was defined as neither wasted nor stunted (HAZ and WHZ >-2 z-scores). To evaluate the intensity of infection, parasite densities were normalized by log-transformation (log (x +1)) and geometric means and 95% confidence intervals (CI) of parasite densities were calculated in each group of children.

Statistical analyses were done using Graph-Pad Prism software (GraphPad, San Diego, CA) and SAS version 8.2 software (SAS, V8.2; SAS Institute, Cary, NC). Geometric means of parasite densities were compared between groups using the paired t-test. The difference between groups in prevalence of immune responders was assessed using the MacNemar test.

Logistic multivariate regressions were used to estimate the association between prevalence of immune responders and malnutrition adjusted for potential confounders (intensity of infection and groups of age).

After verifying that values did not assume a Gaussian distribution, the correlation between IgG Ab levels (log OD) and intensity of infection was estimated using the spearman correlation (non parametric correlation). The Wilcoxon rank signed test (non-parametric match-paired test) was used for comparison of IgG Ab levels between cases and controls.

Generalized linear regressions were used to estimate the association between IgG Ab levels (log OD) and malnutrition adjusted for intensity of infection and groups of age.

All differences were considered significant at *P *< 0.05.

## Results

A total of 322 children were included in this cross-sectional sub-study: 161 were malnourished (either stunted or wasted) and 161 were pair-matched well-nourished controls (Table 1). Among these 161 malnourished children, 142 were stunted and 19 were wasted.

**Table 1:** Characteristics of rural Senegalese preschool children included in the analysis.

	**All children** **n = 322**		**Malnourished children** **n = 161**		**Pair-matched control children** **n = 161**	
Variable	n or mean	% or SD	n or mean	% or SD	n or mean	% or SD
Child's age (months)						
12–23.9	32	19.9	32	19.9	32	19.9
24–35.9	31	19.3	31	19.3	31	19.3
36–47.9	43	26.7	43	26.7	43	26.7
48–60	33	20.5	33	20.5	33	20.5
>60	22	13.6	22	13.6	22	13.6
Mean age (months)	41.4	15.9	40.9	16.0	41.9	15.8
Sex						
Female	166	51.5	83	51.5	83	51.5
Male	156	48.5	78	48.5	78	48.5
Nutritional status						
Height-for-age (z-score)	-1.55	1.04	-2.31	0.72	-0.79	0.71
Weight-for-height (z-score)	-0.42	0.97	-0.61	1.01	-0.22	0.89
Prevalence of malnutrition						
Stunting	142	44.1	142	88.2	0	-
Wasting	19	5.9	19	11.8	0	-

### Correlation between IgG Ab levels and intensity of infection

It has been previously shown that Ab levels directed to wide antigens of *P. falciparum*, such as schizont antigens, were associated with *P. falciparum *infection [29]. Therefore, the intensity of infection could be a confounder in the association between malnutrition and anti-*P. falciparum *Ab response, i.e. IgG Ab levels could be correlated to parasitaemia. The specific *P. falciparum *IgG Ab levels, appeared to be positively correlated with the intensity of infection both among malnourished and control children (r = 0.53, P < 0.0001 and r = 0.52, P < 0.0001, respectively). This positive significant correlation was similar both among stunted children, mildly or severely stunted children and their controls. Among wasted children the correlation seemed to be lower than in the other groups of children, but this correlation was not statistically significant (r = 0.25, P = 0.32).

### The specific anti-*P. falciparum *IgG response according to malnutrition

The prevalence of anti-malarial immune responders (Table 2) was significantly lower in malnourished children (with stunted children and wasted children pooled) than in controls (P = 0.001) whereas no significant difference in the geometric mean of parasite density was observed between the two groups. When adjusting for parasite density using a logistic multivariate regression, the odds ratio of being immune responder was significantly below 1 (OR = 0.4, P = 0.01), indicating that prevalence of immune responders was significantly lower in malnourished children compared to their controls regardless of intensity of infection.

**Table 2:** Specific IgG immune responders and geometric mean of parasite density in rural preschool Senegalese children

	n	Responders ^a ^(%)	P ^b^	n	Geometric mean of parasite density^c ^[95% CI]	P ^d^
Groups of children						
Malnourished children	161	118 (73.3)	0.001	140	3.87 [2.42–6.21]	0.34
Paired controls	161	140 (87.0)		140	5.27 [3.38–8.23]	
						
Stunted children	142	104 (73.2)	0.001	123	4.57 [2.69–7.75]	0.56
Paired controls	142	125 (88.0)		123	5.62 [3.48–9.08]	
						
Wasted children	19	14 (73.7)	0.65	17	1.18 [0.83–1.66]	0.13
Paired controls	19	15 (78.9)		17	3.34 [0.91–12.23]	
						
Stunted children (-2.5<HAZ<-2.0)	84	62 (73.8)	0.01	72	6.44 [3.01–13.80]	0.89
Paired controls	84	74 (88.1)		72	6.28 [3.23–12.22]	
						
Stunted children (HAZ≤-2.5)	58	42 (72.4)	0.03	51	2.71 [1.35–5.43]	0.19
Paired controls	58	51 (87.9)		51	4.93 [2.43–10.02]	

^a ^Number of IgG anti-schizont immune responders

^b ^The prevalence of immune responders between groups was compared using the Mac Nemar test.

^c ^Assessment of parasitaemia missing for 21 pairs of children.

^d ^Geometric means between groups were compared using paired t-test.

**Figure 1:**
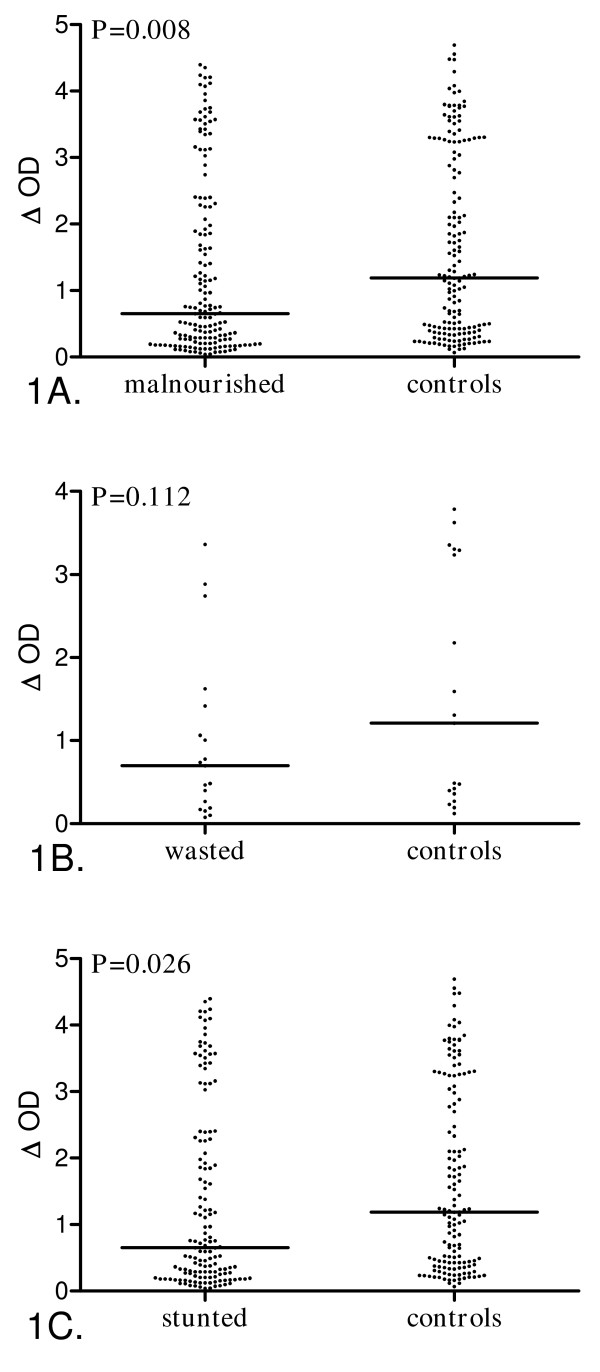
**Anti-*P. falciparum *IgG levels according to malnutrition in rural Senegalese preschool children**. Individual ΔOD are presented and bars indicate the median value for each group. **1A**, Malnourished children (n = 161, both stunted (HAZ<-2) and wasted children (WHZ<-2)) *vs *controls (n = 161, neither stunted nor wasted children). **1B**, Wasted children (n = 19, WHZ<-2) *vs *controls (n = 19, neither stunted nor wasted children). **1C**, Stunted children (n = 142, HAZ<-2) *vs *controls (n = 142, neither stunted nor wasted children). Statistical significance between groups is indicated (Wilcoxon signed rank test).

IgG Ab levels directed to schizont extracts presented according to child malnutrition are presented in Figure 1. A 45% decrease in the IgG Ab level was observed in malnourished children compared to control children (Figure 1A, P = 0.008).

When adjusting for intensity of infection using a generalized linear regression, malnourished children presented a significant 33% decrease of IgG Ab levels compared to well-nourished control children (r = -0.33, P = 0.004).

The specific IgG response was explored according to the type of child malnutrition (Figure 1B, C). The prevalence of immune responders (Table 2,), the IgG Ab levels (Figure 1B), and geometric means of parasite densities (Table 2) tended to be lower in wasted children than in controls, but the difference was not statistically significant. Moreover no significant correlation was found between IgG Ab levels and parasite density among wasted children.

Conversely, the prevalence of immune responders (Table 2) and the specific IgG Ab levels (Figure 1C) were significantly lower in stunted children compared to controls. In contrast, no significant difference was observed in the geometric means of parasite density (Table 2). This lower specific Ab response among stunted children remained significant when adjusting for parasite density, both in terms of prevalence of immune responders (OR = 0.37, P = 0.01) and IgG Ab levels (r = -0.33, P = 0.009). The decrease in anti-*P. falciparum *IgG Ab response observed among stunted children was similar to the one observed among malnourished children.

### The specific anti-*P. falciparum *IgG response according to severity of stunting

**Figure 2:**
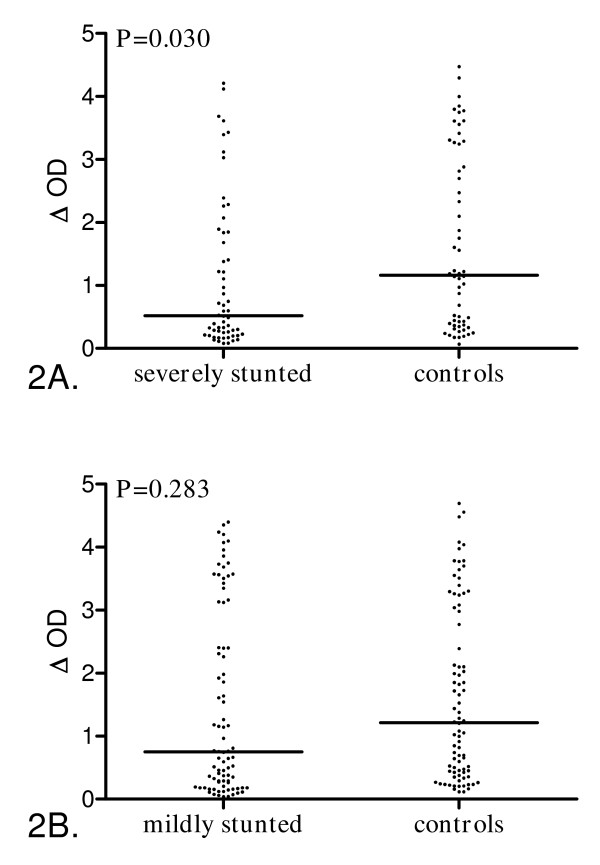
**Anti-*P. falciparum *IgG levels according to height-for-age z-score of stunted preschool children**. Individual ΔOD are presented and bars indicate the median value for each group. **2A**, Mildly stunted children (n = 84, -2.5 < HAZ <-2.0) *vs *controls (n = 84, children with HAZ and WHZ ≥-2). **2B**, Severely stunted children (n = 58, HAZ ≤ -2.5) *vs *controls (n = 58, children with HAZ and WHZ ≥ -2). Statistical significance between groups is indicated (Wilcoxon signed rank test).

The second step involved estimating whether severity of stunting influences the anti-malarial IgG response. Specific IgG Ab levels were compared between stunted children and their pair-matched controls according to mild or severe stunting (Figure 2). Among the 161 stunted children, 58 were severely stunted (HAZ ≤ -2.5) and 84 were mildly stunted (-2.5 < HAZ <-2.0).

The prevalence of immune responders was significantly lower in mildly stunted children than in their controls (Table 2) whereas no significant difference in the geometric mean of parasite density was observed between the two groups.

The specific IgG Ab level was lower among mildly stunted children compared to their controls but this difference was not statistically significant (Figure 2A, P = 0.28). These findings were not adjusted for parasite density because all parasitaemic children among those mildly stunted and controls were immune responders leading to a complete separation of data points. Consequently, in this case the multivariate model was not applicable.

The severely stunted children appeared to be less immune responders compared to their controls (Table 2) whereas no significant difference was found in geometric means of parasite density between the two groups. However this lower prevalence of immune responders among severely stunted children was not statistically significant when adjusting for intensity of infection. A significant 55% decrease in IgG Ab level was found in severely stunted children compared to their pair-matched controls (Figure 2B, P = 0.030), but this decrease did not remain significant when adjusting for intensity of infection.

## Discussion

The main objective of this cross-sectional study conducted in July 2003 at the onset of the rainy season was to investigate whether child malnutrition could modulate the anti-*P. falciparum *Ab response among preschool children.

The findings show that both the prevalence of immune responses and anti-*P. falciparum *IgG Ab levels were significantly lower in malnourished children than in controls regardless of the intensity of infection. Depending on the type of malnutrition, wasted children and stunted children presented a lower specific IgG Ab response than their respective controls, but this difference was only significant in stunted children. This lower specific IgG Ab response remained significant when adjusting for intensity of infection. However, the small number of wasted children was a limitation to this study. Among stunted children, results indicated that the specific IgG response was significantly lower in severely stunted children (HAZ ≤ -2.5) compared to their controls, while no significant difference was observed in mildly stunted children (-2.5 < HAZ <-2.0). However this difference among severely stunted children did not remain significant when adjusting for parasite density. Altogether, these results suggest that malnutrition, and particularly chronic malnutrition (stunting), could down-regulate anti-*P. falciparum *Ab responses in preschool children. Moreover this down-regulation appeared to be independent of the intensity of infection.

Numerous studies have investigated the relationship between child malnutrition and either malaria morbidity or intensity of infection [16,18,20,21]. In contrast, only a few studies have explored the interaction between child malnutrition and specific anti-*P. falciparum *immune responses. Moreover, results of those studies are conflicting [26-28]. Indeed, no association was found between the specific Ab level and acute or chronic undernutrition in Tanzanian children [28], whereas the anti-malarial Ab response was lower in malnourished Colombian children [26] and wasted Papua New Guinean children [15] than in well-nourished children. Several discrepancies between the studies could explain these conflicting results. First, child malnutrition was evaluated using different definitions. Indeed, in the Tanzanian study, children were classified as acutely undernourished based on reduced weight-for-age and normal height-for-age, whereas chronically undernourished children had normal height-for-weight and reduced weight-for-age. In the Colombian study, child malnutrition was defined according to the Waterlow classification (based on weight-for-age and height-for-age [39]) or the Gomez classification (based only on weight-for-age [40]). In a more recent longitudinal study in Papua New Guinea [15], children were classified as stunted, wasted or underweight according to the WHO/NCHS reference [38]. These studies also differ in the age range of the children: from 10 to 120 months in Papua New Guinea and under six years of age in the Colombian study, whereas in Tanzania, subjects were school children. It is generally agreed that active growth faltering occurs mainly during the first year of life [41].

Consequently, the influence of stunting on acquired anti-malarial immunity may be higher in the youngest children during the period of active growth faltering. In addition, those studies were conducted in areas with differing patterns of malaria transmission. This could also explain inconsistencies in results, since acquired specific immunity is closely related to the malaria transmission pattern and the age of infected children [42,43].

The specific IgG response to schizont extracts is frequently associated with *P. falciparum *infection in terms of intensity of infection [29,44,45]. These present findings confirm the association between IgG Ab levels and intensity of infection. Indeed, a significant positive correlation between IgG Ab levels and intensity of infection was observed in all groups of children, excepted for wasted children probably due to the low number of subjects. The lower immune response both in terms of prevalence of immune responders and IgG Ab levels observed among malnourished children remained significant when adjusting for the confounding effect of intensity of infection. In the same way, stunted children presented a significant lower anti-*P. falciparum *Ab response compared to their controls. Considering the degree of stunting, the results suggest that specific Ab response was lower among severely stunted children than in controls children, while no significant difference was found in mildly stunted children. Although this lower Ab response was not significant when adjusting for intensity of infection, these findings nevertheless suggest that severely stunting might down-regulate the anti-*P. falciparum *IgG Ab response.

While, others elements such as genetic factors, different history of malaria infection or self-medication might also explain the differences observed in specific Ab response, the present results indicate that malnutrition, and more precisely stunting, seems to be the most likely cause of down-regulation of the anti-*P. falciparum *IgG response.

Indeed, the childhood nutritional status could regulate development of the acquired protective immune response to malaria antigens. It is now widely admitted that undernutrition during critical periods of childhood growth impairs normal development of the immune system [22]. Malnutrition causes atrophy of the thymus and other lymphoid tissues, reduced B-cell activation and complement formation. Consequently, both acquired immunity and innate host defense mechanisms are affected in malnourished children [24]. Moreover, many studies highlighted the role of micronutrients in host resistance to infection [22,23]. Deficiencies in some micronutrients such as vitamin A, zinc or iron are thought to play an important role in modulation of malaria morbidity; however, little is known about the interaction between micronutrient deficiencies and specific malaria immune responses.

Numerous studies have demonstrated the role of specific IgG isotype responses in anti-malarial protective immunity [30,46,47]. It is generally agreed that cytophilic IgG1 and IgG3 isotypes participate in specific protective immunity, whereas IgG2/IgG4 isotypes block its effect [48]. Malnutrition could regulate the balance between these protective/blocking isotype responses and consequently play a key role in the development of the protective anti-malarial immune response acquired during infection. This hypothesis is currently under investigation.

In addition, nutritional status could modulate the immune response directed to malaria antigens, in particular, to major vaccine candidates [49]. Indeed, a previous study showed that IgG Ab levels against RESA and Spf66 antigens were lower in wasted children compared to well-nourished children [15]. Therefore, future phase 2/3 vaccine trials including major candidates should take into account child nutritional status when evaluating the specific immune response acquired after immunization.

## Conclusion

The main findings of this study indicate that malnutrition may modulate the overall anti-*P. falciparum *IgG Ab response. Moreover, this modulation seems to vary according to the type of malnutrition considered, since stunting, but not wasting, induced down-regulation of the IgG Ab response among preschool children. These results underscore the importance of further understanding how malnutrition influences anti-malarial immunity and subsequent malaria morbidity. With this aim, a specific protective immune response must be investigated according to malnutrition especially during anti-malarial vaccine trials. In addition, the present report highlights the need to take into account child nutritional status in epidemiological studies on malaria.

## Competing interests

The authors declare that they have no competing interests.

## Authors' contributions

All authors read and approved the final manuscript.

FF contributed to conception of the study, statistical analysis and interpretation of data, and drafted the manuscript. JBS contributed to conception of the study and carried out ELISA assessments. DB contributed to database management and helped to the draft manuscript.

BC and CS contributed to conception of the study and field activities. GR, KBS and FR participated in the conception and coordination of the study and helped to draft the manuscript.

The study was supported by the Gates Malaria Partnership, which receives support from the Bill and Belinda Gates Foundation, the LSHTM DFID Malaria Knowledge Programme, the «Institut de Recherche pour le Développement» and by the «Espoirs pour la santé» NGO.

Florie Fillol is supported by a scholarship from the French Ministry of Research.
